# Activation of the Complement/Lectin Pathway, Angiopoietin/Tie‐2/VEGF‐System, Cytokines and Chemokines in Different Angioedema Subtypes

**DOI:** 10.1002/eji.70010

**Published:** 2025-07-20

**Authors:** Katharina Marlies Duda, Manuela Gehring, Bettina Wedi

**Affiliations:** ^1^ Department of Dermatology and Allergy Comprehensive Allergy Center Hereditary Angioedema Center for Rare Diseases Hannover Medical School Hannover Germany

**Keywords:** angioedema, biomarkers, chemokines, clinical immunology, complement, eosinophils, granulocytes, hereditary angioedema, inflammation, lectin

## Abstract

The precise molecular mechanisms underlying angioedema attacks in cases of C1‐inhibitor‐deficient hereditary angioedema (C1‐INH‐HAE), angiotensin‐converting enzyme inhibitor‐induced angioedema (ACEi‐AE), and mast cell‐/histamine‐mediated angioedema (Hist‐AE) are not well understood. These attacks may involve immune and inflammatory mechanisms. We compared serum biomarkers indicating vascular integrity, leakage, angiogenesis, coagulation, and inflammation. During an attack‐free period, we assessed 34 markers simultaneously using multi‐ and/or singleplex ELISA in 25 patients with C1‐INH‐HAE, 17 with ACEi‐AE, 25 with Hist‐AE, and 23 healthy controls. Differential blood counts, C1‐INH‐HAE‐specific laboratory parameters, and recently developed assays addressing early complement and lectin pathways were included. The results revealed significant differences, as well as some similarities. Tie‐2, VEGFs, C1s/C1INHc appear to play a role in all AE types regardless of whether they are bradykinin‐ or histamine‐mediated. Furthermore, evidence was found for a role of IL‐17, eosinophil, and neutrophil chemotactic factors, and the activation of these granulocytes was found. MASP‐1/C1‐INHc indicated early activation of the lectin pathway in ACEi‐AE and HistAE, but not in C1‐INH‐HAE. C1s/C1‐INHc and MASP‐1/C1INHc ratio was able to discriminate C1‐INH‐HAE from controls and the other AE types. Future investigations in C1‐INH‐HAE, ACEi‐AE, and Hist‐AE should not only focus on complement activation but also the interaction with granulocytes.

## Graphical abstract

Biomarkers of vascular integrity and inflammatory processes are involved in different types of angioedema, highlighting the potential importance of complement/lectin system interactions with granulocytes in understanding angioedema triggers. The C1s/C1‐INHc‐to‐MASP‐1/C1‐INHc ratio could be used as a diagnostic tool to distinguish C1‐INH‐HAE from other HAE subtypes.

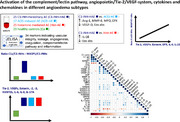

**Abbreviations**
ACEi‐AE: angiotensin‐converting enzyme induced angioedema
AE: angioedema
Ang‐1: human angiopoietin 1
Ang‐2: human angiopoietin 2
C1‐INH: C1 inhibitor
C1‐INHc: C1 esterase inhibitor complex
C1s: complement component 1s
C3a: complement component 3a
CSU: chronic spontaneous urticaria
DPPIV: dipeptidylpeptidase IV
EETs: eosinophil extracellular traps
EPX: human eosinophil peroxidase
HAE: hereditary angioedema
Hist‐AE: histamine‐mediated angioedema
HMWK: high molecular weight kininogen
IgE: immunoglobulin E
IL: interleukin
ITIH4: inter‐α‐trypsin inhibitor heavy chain 4
MASP‐1: Mannan‐binding lectin‐associated serine protease‐1
MMP‐9: matrix‐metalloproteinase
MPO: myeloperoxidase
MRGPRX2: Mas‐related G‐protein coupled receptor member X2
NETs: neutrophil extracellular traps
RANTES: regulated and normal T cell expressed and secreted
Spk‐1: Sphingosinkinase 1
Tie‐2: tyrosine kinase with immunoglobulin and EGF homology domains 2
TIMP‐1: tissue Inhibitor of Metalloproteinases 1
tPA: tissue plasminogen activator
uPAR: urokinase plasminogen activator surface receptor
VEGF: vascular endothelial growth factor
vWF: von Willebrand Factor

## 1 Introduction

Angioedema (AE) is characterized by recurrent episodes of swelling in subcutaneous or submucosal tissues, devoid of pruritus or hives [1, 2]. The most common localizations are the extremities, the abdomen, and the upper respiratory tract. It is important to note that AEs can be indicative of a wider range of underlying pathologies. On the one hand, genetic mutations that affect the complement system (C1‐inhibitor deficiency) and result in bradykinin excess can be the cause (C1‐INH‐HAE). Conversely, medications such as ACE inhibitors, which result in an increased amount of bradykinin by inhibiting the renin–angiotensin system, can act as triggers (ACEi‐AE). Furthermore, the release of histamine from mast cells, for example, in the context of chronic spontaneous urticaria, can also lead to angioedema (Hist‐AE).

The primary cause of C1‐INH‐HAE is attributed to deficiency (type 1) or dysfunction (type 2) of C1 inhibitor (C1‐INH), a protein that is a major natural inhibitor of kallikrein and Factor XIIa [1]. The etiology of this condition is the result of elevated bradykinin production, which subsequently activates bradykinin B2 receptors. This, in turn, leads to vascular permeability being disrupted, resulting in endothelial leakage and angioedema [1, 3]. C1‐INH exerts a regulatory influence over the kallikrein‐kinin system, complement and fibrinolytic pathways, as well as contact activation and the coagulation system [4]. For instance, the absence of C1‐INH results in the autoactivation of C1r, which subsequently leads to the activation of C1s, followed by C4 cleavage and depletion [5]. The significant clinical variation in disease severity observed among family members carrying the same causative mutation remains to be explained. Furthermore, the enigma of systemic prodromes antedating attacks with local manifestations by a few hours or even longer remains unresolved [7, 8]. A common prodrome is a non‐pruritic skin rash that can be misdiagnosed as urticaria (*Erythema marginatum*) [6].

It is hypothesized that the effect of ACEi‐AE is mediated by an increase in local bradykinin, consequent to pharmacological ACE blockade [7]. A recent genome‐wide association study has led to the identification of further risk loci in ACEi‐AE. The results obtained suggest that not only the BK signaling and coagulation pathways but also the fibrinolysis pathway are involved [8]. As demonstrated in the variance study, approximately 70% of ACEi‐AE occurrence, which manifests only after a considerable delay of months to years following the commencement of treatment, remains a subject of intrigue [9]. This delay in onset, in addition to its sporadic nature, further complicates the understanding of the underlying mechanisms.

In the context of chronic spontaneous urticaria, the pathophysiology of AE is characterized by mast‐cell/histamine‐mediated reactions [2] with the potential for underlying autoimmune mechanisms [10]. It is well established that there is a role for peripheral blood basopenia, eosinopenia [10], and coagulation and complement factors [11, 12]. In addition, there is a paucity of evidence for bradykinin release [7].

Despite the pathophysiology of these two conditions being regarded as distinct, there are some common features shared by bradykinin‐ and histamine‐mediated AEs. Notwithstanding, the manifestation of angioedema attacks is erratic with regard to timing and location. Triggers such as trauma, stress, infections, and inflammation have been associated with these attacks, although the exact mechanism is not fully understood [13]. Although they are clinically indistinguishable, angioedema subtypes require markedly different emergency treatments [1, 2].

Asymptomatic intervals are known to be subject to interruption by attacks, and it is therefore hypothesized that these attacks may involve immune and inflammatory mechanisms. In a recent study, several potential biomarkers were identified as a means to differentiate between bradykinin‐ and mast cell‐/histamine‐mediated AE [14]. The objective of this study was to validate the previously described biomarkers, including Tie‐2, using a larger number of subjects. Furthermore, the study incorporated markers of coagulation, vascular integrity, angiogenesis, inflammation, and immune cells.

## 2 Material and Methods

### 2.1 Patients and Blood Samples

In total, 25 patients with C1‐INH‐HAE (24 with type I, one with type II) aged 18 to 76 years, 17 patients with Angiotensin converting enzyme inhibitor‐induced AE (ACEi‐AE), 25 patients with mast‐cell/histamine‐induced angioedema in chronic spontaneous urticaria without wheals (Hist‐AE), and 23 healthy controls were included. All participants identified themselves as of European descent. Serum samples and clinical data were prospectively collected after signed informed consent (ethics committee approval no. 7409, Hannover Medical School). There was no significant difference regarding age or sex between the groups (data not shown). Diagnostic criteria, the questionnaires, blood sample collection, and routine laboratory parameters were used as described earlier [14].

### 2.2 Immunoassays for Potential Biomarkers

C1s/C1‐INH complex (Hycult Biotech, Uden, the Netherlands) for classical pathway, and MASP‐1/C1‐INH complex (Hycult Biotech) for early lectin pathway activation were used. Human angiopoietin (Ang‐) 1 and 2, tyrosine kinase with immunoglobulin and epidermal growth factor homology domains 2 (Tie‐2), urokinase plasminogen activator surface receptor (uPAR), Pentraxin‐3, dipeptidylpeptidase IV (DPPIV), tissue plasminogen activator (tPA), von Willebrand factor (vWF), complement (C) 3a, vascular endothelial growth factor (VEGF)‐A, VEGF‐C, VEGF‐D, Eotaxin (CCL26), Eotaxin‐2 (CCL24), Eotaxin‐3 (CCL26), regulated and normal T cell expressed and secreted (RANTES, CCL5), interleukin (IL‐) 1ß, 6, 8 (CXCL8), 10, 17A, 23, myeloperoxidase (MPO), matrix‐metalloproteinase 9 (MMP‐9), granzyme B, Sphingosinkinase 1 (Spk‐1), galectin‐3, tissue inhibitor of metalloproteinases 1 (TIMP‐1), immunglobulin E (IgE) were assessed by using multiplex ELISA from MSD (Meso Scale Discovery Inc., Rockville, MD, USA), encompassing the following subtypes: V‐Plex (validated single and multiplex assays), U‐Plex (flexible, customized multiplex assays) and R‐Plex (matched antibody sets for building own multiplex assays). For human eosinophil peroxidase (EPX) and Mas‐related G‐protein coupled receptor member X2 (MRGPRX2), the ELISAs manufactured by MyBiosource (San Diego, CA, USA) were used. Additionally, IL‐17A was determined using the single‐plex DuoSet IL‐17 bio‐techne R&D Systems (Minneapolis, USA). All measurements were performed in duplicate in sera according to the manufacturer's recommendations and our own pilot experiments. On each microtitre plate, serum samples from controls and all angioedema subtypes were equally distributed.

### 2.3 Statistics

For data analysis, R‐Studio (R version 4.0.2) and GraphPad Prism Version 9 (Software, La Jolla, California, USA) were used. Quantile–quantile (q–q) plot and a Shapiro–Wilk test were used to test the normality of the data distribution. Statistical significance was determined by two‐tailed *t*‐test or Wilcoxon test; ANOVA with the post hoc Tukey test or Kruskal–Wallis test where appropriate. *p*‐values <0.05 were considered statistically significant. For the calculation of correlation coefficients and data visualization corrplot package was used [15]. CorelDraw (Alludo, Canada) was used for visualization of the figures.

A single patient with C1‐INH‐HAE type II who had a normal C1‐INH concentration was excluded from the statistical analysis if it concerned the C1‐INH concentration.

## 3 Results

### 3.1 Classical and Lectin Complement Pathway

**FIGURE 1:**
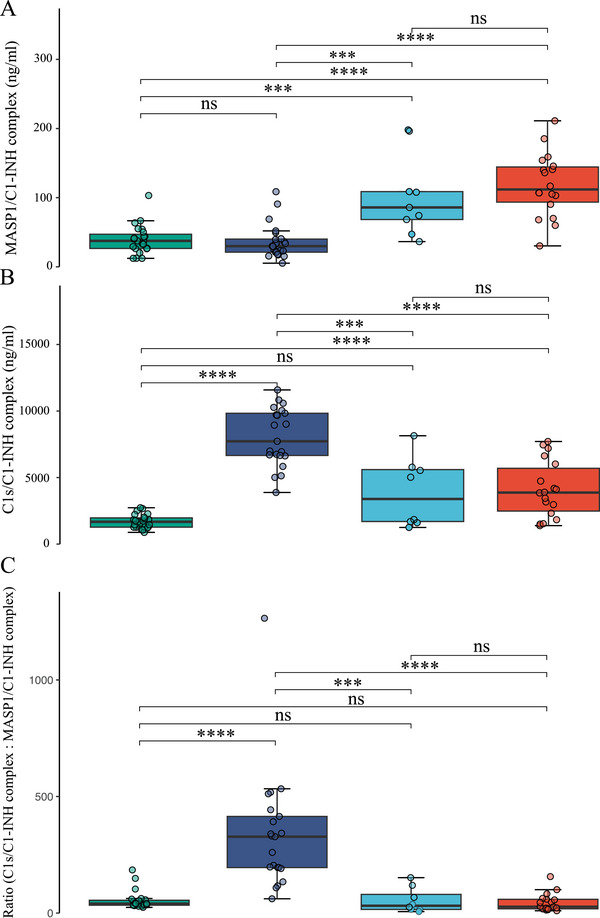
**Analysis of the classical and lectin pathway of the complement system in healthy donors, C1‐INH‐HAE, ACEi‐AE, and Hist‐AE**. Box‐and‐whisker plots show serum levels of mannan‐binding lectin‐associated serine protease 1 (MASP1)/C1‐INH complex (Panel A), complement component 1s (C1s)/C1INH complex (Panel B), and the ratio of both (Panel C). Healthy controls (green, *n* = 23), C1‐INH‐HAE patients (dark blue, *n* = 30), ACEi‐AE patients (light blue, *n* = 17), and Hist‐AE patients (red, *n* = 25). Unpaired Wilcoxon test; ***, *p *< 0.001; ****, *p *< 0.0001; ns, not significant.

To discern the specific complement pathway (either classical or lectin) involved, MASP1/C1‐INH‐ and C1s/C1‐INH complexes were measured. MASP1/C1‐INH complexes were similar in healthy controls and C1‐INH‐HAE, but significantly elevated in ACEi‐AE and Hist‐AE compared with controls and C1‐INH‐HAE (Figure 1A). Conversely, C1s/C1‐INH complexes were found to be statistically significantly increased in C1‐INH‐HAE sera compared with healthy controls (Figure 1B, *p* < 0.0001), ACEi‐AE (*p* < 0.001), and Hist‐AE (*p* < 0.0001). C1s/C1‐INH complexes in C1‐INH‐HAE were even significantly higher compared with ACEi‐AE and Hist‐AE. We did not find any sex‐related differences regarding MASP1/C1‐INH‐ and C1s/C1‐INH complexes (not shown). The ratio of C1s/C1‐INH complexes: MASP1/C1‐INH complexes was significantly elevated in C1‐INH‐HAE only (Figure 1C, *p* < 0.0001), thereby providing an additional discriminating in vitro parameter.

### 3.2 Comparative Analysis of Regulators of Vascular Integrity, Leakage, and Angiogenesis

Serum levels of Tie‐2, VEGF‐A, VEGF‐C, and VEGF‐D were increased in all AE subtypes compared with healthy controls (Figure 2A, *p* < 0.0001). There were no statistically significant differences among the AE subtypes, except for VEGF‐D, which was significantly higher in ACEi‐AE compared with C1‐INH‐HAE (*p* < 0.05). Serum Ang‐1 levels were higher in C1‐INH‐HAE compared with controls (*p* < 0.01) and compared with ACEi‐AE (*p* < 0.05). MMP‐9 was significantly increased in C1‐INH‐HAE compared with controls (*p* < 0.01) and compared with ACEi‐AE (*p* < 0.05), but not compared with Hist‐AE. In contrast, TIMP‐1, representing the main inhibitor of MMP‐9, did not show abnormalities (not shown). In contrast, the levels of Ang‐2, Pentraxin‐3, DPPIV, uPAR, vWF, C3a (all in Figure 2A), and granzyme B, galectin‐3, and SPK‐1 (data not shown) were similar in all AE subtypes and controls.

FIGURE 2(A) Comparative analysis of parameters reflecting vascular integrity, leakage, and angiogenesis in healthy controls, C1‐INH‐HAE, ACEi‐AE, and Hist‐AE. Serum levels of Tie‐2, VEGF‐A, VEGF‐C, VEGF‐D, Ang‐1, Ang‐2, Pentraxin‐3, DPPIV, uPAR, vWF, C3a, and MMP‐9 in healthy controls (green, *n* = 23), C1‐INH‐HAE (dark blue, *n* = 30), as well as in ACEi‐AE (light blue, *n* = 17) and Hist‐AE (red, *n* = 25), respectively. Unpaired Wilcoxon test; *, *p *< 0.05; **, *p *< 0.01; ***, *p *< 0.001; ****, *p *< 0.0001; ns, not significant. (B) **Comparative analysis of parameters reflecting chemokines and cytokines in healthy controls, C1‐INH‐HAE, ACEi‐AE, and Hist‐AE**. Serum levels of MPO, EPX, Eotaxin, Eotaxin‐2, Eotaxin‐3, RANTES, IL‐1beta, IL‐6, IL‐8, IL‐10, IL‐17A, IL‐23 in healthy controls (green, *n* = 23), C1‐INH‐HAE (dark blue, *n* = 30), as well as in ACEi‐AE (light blue, *n* = 17) and Hist‐AE (red, *n* = 25), respectively. Unpaired Wilcoxon test; *p < 0.05; **p < 0.01; ***p < 0.001; ****p < 0.0001; ns, not significant.

**Figure:**
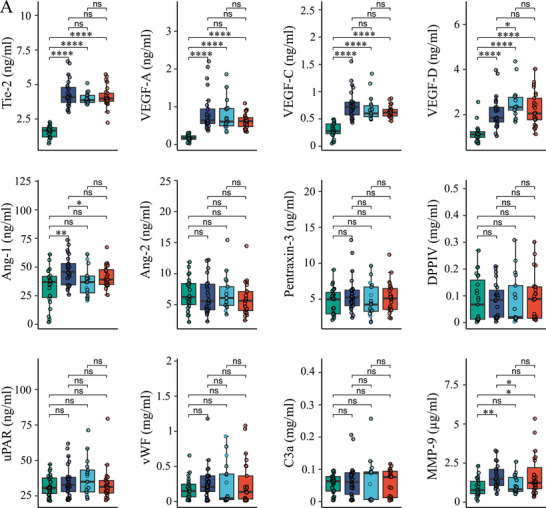

**Figure:**
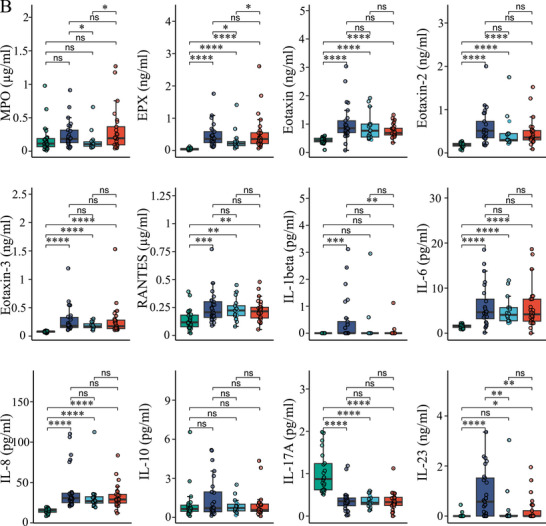

### 3.3 Comparative Analysis of Chemokines and Inflammatory Cytokines

Several chemokines and inflammatory cytokines were significantly elevated in all AE subtypes compared with controls (Figure 2B): EPX (*p* < 0.0001), Eotaxin (*p* < 0.0001), Eotaxin‐2 (*p* < 0.0001), Eotaxin‐3 (*p* < 0.0001), RANTES (*p* < 0.01, *p* < 0.001, respectively), IL‐6 (*p* < 0.0001), and IL‐8 (*p* < 0.0001). In contrast, IL‐17A was significantly lower in all AE types compared with controls (*p* < 0.0001), whereas IL‐23 levels were remarkably higher in C1‐INH‐HAE compared with controls (*p* < 0.0001), ACEi‐AE (*p* < 0.01), and Hist‐AE (*p* < 0.01). This divergent behavior of IL‐17A and IL‐23 was unexpected, therefore we confirmed our multiplex measurements by using a second single‐plex ELISA system and found a considerably positive correlation of both assays for IL‐17A (*R* = 0.96, *p* < 2.2e‐16, not shown) and for IL‐23 (*R* = 0.7, *p* = 1.2e−14, not shown). Furthermore, in ACEi‐AE, MPO and EPX levels were notably lower compared with Hist‐AE (*p* < 0.01) and C1‐INH‐HAE (*p* < 0.05). IL‐1ß was significantly increased in C1‐INH‐HAE compared with controls (*p* < 0.001) and compared with Hist‐AE (*p* < 0.01), but not compared with ACEi‐AE. In contrast, IL‐10, IgE, and MRGPRX2 levels were similar in healthy controls and all AE types (data not shown).

### 3.4 Comparison of Differential Blood Counts

**FIGURE 3:**
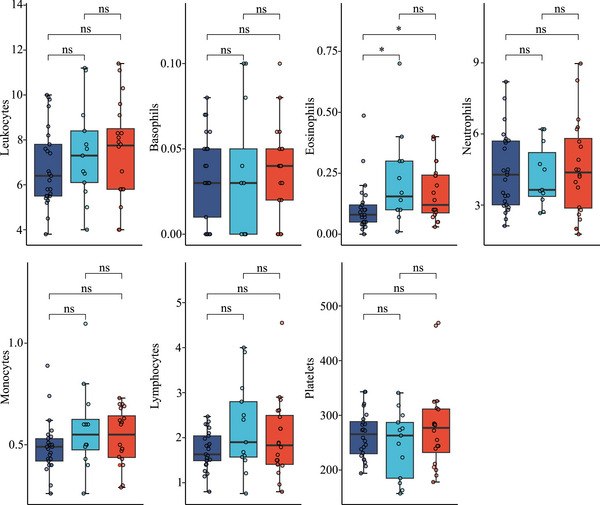
**C1‐INH‐HAE, ACEi‐AE, and Hist‐AE analysis of differential count**. Box plots show serum levels of the following differential counts: leukocytes, absolute values of basophils, eosinophils, neutrophils, monocytes, lymphocytes and platelets in C1‐INH‐HAE (dark blue, *n* = 25), as well as in ACEi‐AE (light blue, *n* = 17) and Hist‐AE (red, *n* = 25), respectively. Unpaired Wilcoxon test; *, *p* < 0.05; ns, not significant.

Differential blood counts were similarly distributed among the AE types (Figure 3) except for absolute eosinophil counts, which were significantly lower in C1‐INH‐HAE compared with ACEi‐AE and Hist‐AE (*p* < 0.05 each).

### 3.5 Correlation Matrix of all Potential Biomarkers

**FIGURE 4:**
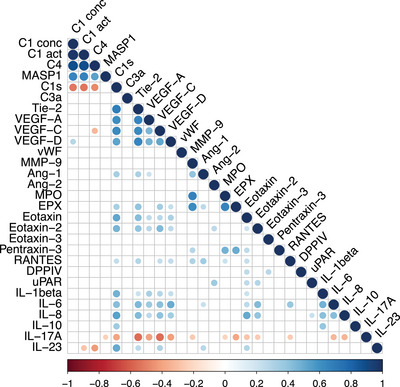
**Correlation matrix of all potential biomarkers (without blood parameters) assessed in all individuals**. Pairwise correlation among various parameters of all individuals assessed. The areas of circles represent absolute values of corresponding correlation coefficients between C1‐INH‐concentration (C1 conc) and C1‐INH‐activity (C1 act), complement C4, MASP1, C1s, C3a, Tie‐2, VEGF‐A, VEGF‐C, VEGF‐D, vWF, MMP‐9, Ang‐1, Ang‐2, MPO, EPX, Eotaxin, Eotaxin‐2, Eotaxin‐3, Pentraxin‐3, RANTES, DPPIV, uPAR, IL‐1beta, IL‐6, IL‐8, IL‐10, IL‐17A, and IL‐23. Correlation coefficients range from −1 (perfect negative correlation, depicted by a darker red color) to 1 (perfect positive correlation, depicted by a darker blue color), with 0 indicating no correlation. Displayed are only data where *p* < 0.05.

To identify associations between the various measured parameters, a correlation matrix was created for the striking parameters (Figure 4). As expected, a robust positive correlation was found between C1‐INH‐concentration and C1‐INH‐activity (*r* = 0.97, *p* < 0.0001), C1‐INH‐concentration (*r* = 0.88, *p* < 0.0001), and C4 concentration (*r* = 0.89, *p* < 0.0001). In addition, MASP1‐/C1‐INH‐complexes were significantly positively associated to C1‐INH‐concentration (*r* = 0.66, *p* < 0.0001), C1‐INH‐activity (*r* = 0.67, *p* < 0.0001), and C4 (*r* = 0.53, *p* < 0.01).

**FIGURE 5:**
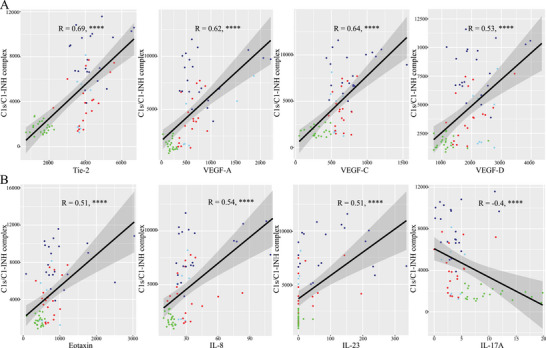
Scatter plots demonstrating correlation of C1s/C1‐INH complex with Tie‐2, VEGF‐A, VEGF‐C, or VEGF‐D, respectively (top row), and of C1s/C1‐INH complex with Eotaxin, IL‐8, IL‐23, and IL‐17A, respectively (bottom row). Healthy controls (green dots), C1‐INH‐HAE (dark blue), ACEi‐AE (light blue), Hist‐AE (red). Pearson correlation coefficients (R) and p values are indicated in the figure, ****p < 0.0001.

In contrast C1s‐/C1‐INH‐complexes were negatively associated to C1‐INH‐concentration (*r* = −0.52, *p* < 0.001), ‐activity (*r* = ‐0.54, *p* < 0.001), C4 (*r* = −0.48, *p* < 0.01), and IL‐17 (*r* = −0.40, *p* < 0.001), and lymphocytes absolute (*r* = −0.36, *p* < 0.05) but positively correlated to Tie‐2 (*r* = 0.69, *p* < 0.0001), VEGF‐A (*r* = 0.62, *p* < 0.0001), VEGF‐C (*r* = 0.64, *p* < 0.0001), VEGF‐D (*r* = 0.88, *p* < 0.0001), Eotaxin (*r* = 0.53, *p* < 0.0001), Eotaxin‐2 (*r* = 0.46, *p* < 0.0001), EPX (*r* = 0.33, *p* < 0.01) IL1‐beta (*r* = 0.48, *p* < 0.0001), IL‐6 (*r* = 0.42, *p* < 0.001), IL‐8 (*r* = 0.53, *p* < 0.0001), and IL‐23 (*r* = 0.51, *p* < 0.0001) (in part shown in Figure 5).

**FIGURE 6:**
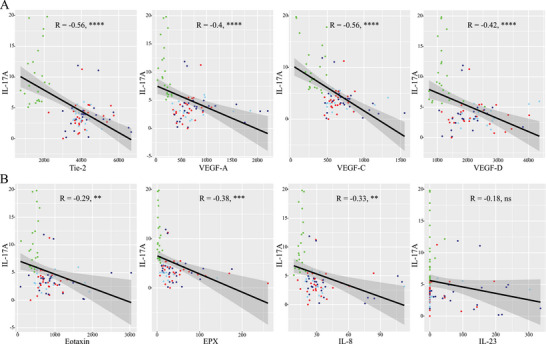
Scatter plots demonstrating correlation of IL‐17A with Tie‐2, VEGF‐A, VEGF‐C, and VEGF‐D, respectively (top row), and of IL‐17A with Eotaxin, EPX, IL‐8, and IL‐23, respectively (bottom row). Healthy controls (green dots), C1‐INH‐HAE (dark blue), ACEi‐AE (light blue), Hist‐AE (red). Pearson correlation coefficients (R) and *p*‐values are indicated in the figure. **p < 0.01; ***p < 0.001; ****p < 0.0001; ns, not significant.

There was a significant negative correlation of IL‐17A to VEGF‐A (*r* = −0.40, *p* < 0.0001), VEGF‐C (*r* = −0.56, *p* < 0.0001), VEGF‐D (*r* = 0.42, *p* < 0.0001), Tie‐2 (*r* = −0.56, *p* < 0.0001), and platelets (*r* = 0.46, *p* < 0.001) but not to C1INH activity, C1‐INH concentration or C4 (in part shown in Figure 6). In addition, we found a significant negative correlation of IL‐17A to Eotaxin (*r* = −0.29, *p* < 0.01), Eotaxin‐2 (*r* = −0.26, *p* < 0.05), EPX (*r* = −0.38, *p* < 0.001) and IL‐8 (*r* = −0.33, *p* < 0.01), but not to IL‐23 (in part shown in Figure 6).

**FIGURE 7:**
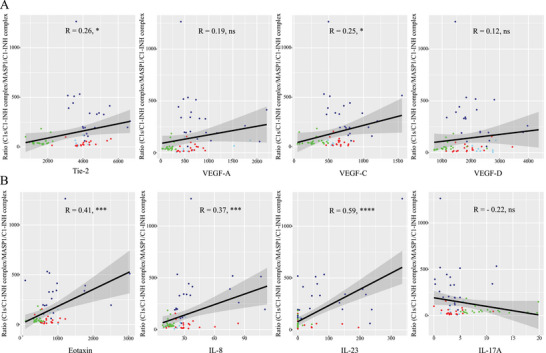
Scatter plots demonstrating the correlation of the Ratio of C1s/C1‐INHc: MASP1/C1‐INHc with Tie‐2, VEGF‐A, VEGF‐C, or VEGF‐D, respectively (top row), and with Eotaxin, IL‐8, IL‐23, and IL‐17A (bottom row). Healthy controls (green dots), C1‐INH‐HAE (dark blue), ACEi‐AE (light blue), Hist‐AE (red). Pearson correlation coefficients (R) and *p*‐values are indicated in the figure. *p < 0.05; ***p < 0.001; ****p < 0.0001; ns, not significant.

Significant positive correlations were found (Figure 7) of the Ratio of C1s/C1‐INHc: MASP1/C1‐INHc with Tie‐2 (*p* < 0.05), VEGF‐C (*p* < 0.05), Eotaxin (*p* < 0.001), IL‐8 (*p* < 0.001), and IL‐23 (*p* < 0.0001), but not to VEGF‐A, VEGF‐D, and IL‐17A.

**FIGURE 8:**
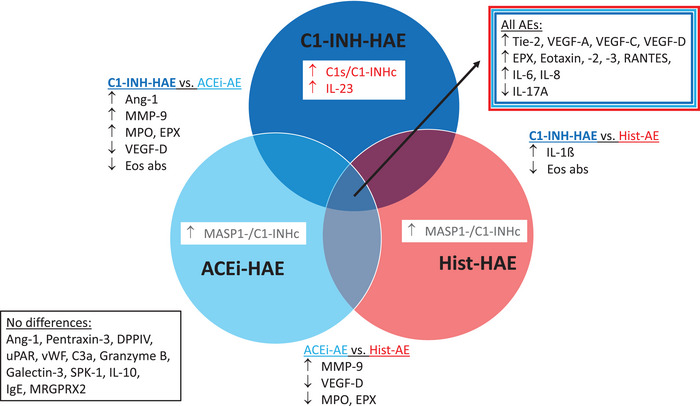
Venn diagram summarizing the overlaps and differences found with regard to the parameters examined in relation to the three types of angioedema.

Figure 8 summarizes the findings in relation to the three angioedema groups.

## 4 Discussion

This study presents novel insights into the activation of the complement and lectin pathways in different AE subtypes using two new commercially available assays. The formation of C1s/C1‐INH complexes occurs as a result of C1‐INH binding with C1r and C1s. Consequently, C1s/C1‐INHc levels were significantly increased in C1‐INH‐HAE owing to C1‐INH dysfunction, in comparison to controls and other AE subtypes (Figure 1). Furthermore, MASP‐1/C1‐INHc and C1s/C1INHc demonstrated a significant correlation with the laboratory parameters that are characteristic of C1‐INH‐HAE (C1‐INH concentration, activity, and C4). Nevertheless, we discovered evidence for elevated C1s/C1‐INHc levels in ACEi‐AE and Hist‐AE for the first time (Figure 1). This finding suggests a substantial activation of the early complement pathway in these AE subtypes. However, this activation was significantly less pronounced compared with that observed in C1‐INH‐HAE. The C1s/C1‐INHc and MASP‐1/C1INHc ratio was found to be a valuable diagnostic tool capable of differentiating C1‐INH‐HAE from both controls and the other AE subtypes (Figures 1 and 8). The C1s/C1‐INHc values measured in healthy individuals are comparable to previously published data by Hurler et al. [16]. While Kajdácsi et al. [17] found no differences between C1‐INH‐HAE patients and controls in a smaller number of subjects, others, including the present authors, have shown higher C1s/C1‐INHc in C1‐INH‐HAE [17, 18, 19] and at times also lower MASP‐1/C1‐INHc levels [20]. MASP‐1 cleaves high molecular weight kininogen (HMWK) into bradykinin, and C1‐INH has been shown to form complexes with MASP‐1 [21]. However, MASP‐1/C1‐INHc levels were found to be elevated in both the C1‐INH‐HAE and healthy control groups. Conversely, MASP‐1/C1‐INHc levels exhibited a marked increase in both ACEi‐AE and Hist‐AE. To the best of our knowledge, this has not been investigated before and indicates an early activation of the lectin pathway in ACE‐iAE and Hist‐AE but not in C1‐INH‐HAE. As demonstrated in Figure 4, a significant positive correlation was identified between C1s/C1‐INHc and Tie‐2, VEGF‐A, VEGF‐C, and VEGF‐D, as well as for IL1ß, IL‐6, IL‐8, and IL‐23. Conversely, a negative correlation was observed with IL‐17A.

A novel missense mutation in *DAB2IP* has recently been reported, resulting in dysregulated VEGF‐mediated signaling and HAE with an unknown mutation [24]. The data presented herein indicate that altered VEGF signaling is evident in all AE types (Figures 2A and 8), irrespective of whether this is mediated by bradykinin or histamine. As VEGFs are well known to directly increase endothelial vascular permeability [23], this suggests a disturbance even during attack‐free intervals. VEGFs have been associated with inflammation, with their release being initiated by neutrophils, for example. In ACEi‐AE, a statistically significant correlation was identified between VEGF‐C and leukocytes, as well as absolute neutrophils.

Confirmation of Tie‐2 as a potential serum biomarker for C1‐INH‐HAE was achieved in comparison to healthy controls (Figure 2A). After our initial description [14], to our knowledge, there have been no further publications regarding Tie‐2 in relation to C1‐INH‐HAE. The subsequent correlation analysis revealed statistically noteworthy correlations between Tie‐2 and the newly added VEGF family members, VEGF‐A and VEGF‐D, and C1s/C1‐INHc (Figure 4). VEGFs and angiopoietins (Angs), the latter bind to Tie‐2, modulate vascular permeability [25]. Loffredo et al. [26] demonstrated increased plasma concentrations of VEGF‐A and VEGF‐C, Ang‐1 and Ang‐2 in C1‐INH‐HAE patients, with these concentrations correlating with disease severity. In contrast to the findings of this study, which demonstrated an absence of association, Loffredo et al. [26] found that VEGF‐A was inversely associated with functional C1‐INH activity. Nevertheless, in the present study, VEGF‐D demonstrated a positive correlation with Tie‐2, as well as with C1‐INH concentration and C4. Ferrara et al. [27] described that plasma concentrations of VEGF‐A, VEGF‐C, and VEGF‐D remained unchanged during the acute phase of the attack compared with the remission phase. Furthermore, no alterations of VEGF‐D were observed during the remission phase compared with controls. Ang1 levels were increased during attacks compared with symptom‐free intervals, while Ang2 levels remained unaltered [27]. Notably, this study provides novel insights by examining elevated levels of VEGF‐A, VEGF‐C, and VEGF‐D in both ACEi‐AE and Hist‐AE (Figures 2A and 8). The present study aims to contribute to the ongoing debate on the role of VEGF levels in chronic spontaneous urticaria (CSU), with and without angioedema [28, 29, 30]. It is evident that none of the aforementioned studies distinguished between patients with and without angioedema, nor did they differentiate between those with angioedema with wheals and those without wheals.

The peptide fragment C3a is an anaphylatoxin able to trigger endothelial cells and activate histamine release from mast cells and basophils [31]. However, no significant differences in C3a levels were observed, nor were any substantial correlations identified with the other parameters evaluated (Figures 2A and 4). Other parameters that are either directly or indirectly implicated in vascular permeability or dysfunction, such as Granzyme B [32, 33] or SPK‐1 [34], were similar across all groups.

Statistically significant higher levels of chemokines that attract neutrophils and eosinophils, namely Eotaxin, Eotaxin‐2, Eotaxin‐3, IL‐8, and RANTES, were found in all AE subtypes (Figures 2B and 8). Additionally, the activation and degranulation marker for eosinophils, namely EPX, was significantly higher in all AEs compared with controls. Conversely, MPO levels did not demonstrate a similar trend. However, both MPO and EPX levels were found to be significantly higher in C1‐INH‐HAE compared with ACEi‐AE, suggesting an activation of neutrophils and eosinophils in the former condition but not the latter (Figures 2B and 8).

Chronic spontaneous urticaria (CSU) is characterized by basopenia and eosinopenia in 10% to 15% of patients [35, 36]. However, the literature does not provide any evidence as to whether this is also a characteristic of Hist‐AE without wheals. In the present study, Hist‐AE was not found to be associated with basopenia or eosinopenia. In contrast, absolute eosinophil counts were lower in C1‐INH‐HAE compared with ACEi‐AE and Hist‐AE (Figures 3 and 8). As demonstrated by others, an increase in the number of neutrophils has been observed in C1‐INH‐HAE during acute attacks, but not at attack‐free intervals [37]. Moreover, during asymptomatic periods, a dysregulation of neutrophils was identified [38]. To the best of our knowledge, no research has hitherto been conducted on the role of eosinophils in C1‐INH‐HAE. In this regard, it may be worth mentioning that the direct role of C1‐INH in leukocyte–endothelial cell adhesion has been demonstrated in several studies [39, 40].

C1‐INH is a crucial regulator of the contact system (also called the plasma kallikrein kinin system), which is constitutively activated in HAE [41, 42]. The results of this study suggest the potential involvement of eosinophil and neutrophil chemotactic factors, as well as granulocyte activation. These phenomena could contribute to the development of angioedema through additional excessive activation of the contact system [43, 44]. Very recent data described inter‐α‐trypsin inhibitor heavy chain 4 (ITIH4) as a compensatory protease inhibitor and potential biomarker in C1‐INH‐HAE [45]. ITIH4 has been found to target both the innate immune and contact systems. It is noteworthy that inhibitory effects of ITIH4 on MASP‐1 and MASP‐2 have been described [45]. Furthermore, ITIH4 has been shown to have the capacity to counteract both eosinophilic and neutrophilic inflammation. For instance, it has been shown to play a role in the migration of neutrophils [46].

IL‐1ß and IL‐6, as well as MMP‐9, the latter being abundant in neutrophils, were particularly increased in C1‐INH‐HAE compared with controls (Figure 2B). A comparison of these results with those from the literature reveals that the elevated serum proteins IL‐8, IL‐1ß, and MMP‐9 correspond with the increased mRNA expression demonstrated by Grymova et al. [38] in neutrophils of C1‐INH‐HAE patients compared with healthy individuals. However, their investigation did not extend to the analysis of these proteins in plasma, and no elevated plasma levels of neutrophil elastase or MPO were detected. In addition, the present study revealed that MPO levels were not significantly increased in C1‐INH‐HAE (Figure 2B).

Vascular endothelial galectins play a role in leukocyte trafficking [47]. Increased serum galectin‐1, galectin‐3, and galectin‐9 levels have been described in chronic spontaneous urticaria [48], and very recently, increased galectin‐9 expression on basophils and eosinophils has been demonstrated [49]. In both studies, the proportion of patients with Hist‐AE without wheals was not described separately. In the context of galectin‐3 serum levels, no evidence was found to suggest a role in C1‐INH‐HAE, ACEi‐AE, and Hist‐AE.

Low total IgE levels have been attributed to a subpopulation of CSU with less response to anti‐IgE treatment [50]. The activation of the multifaceted MRGPRX2 has been described in pseudoallergy and urticaria [51, 52] and very recently was suspected as a missing link between Hist‐AE and C1‐INH‐HAE [53]. However, in the present study, both IgE and MRGPRX2 levels were found to be similar in healthy controls and across all AE types (data not shown).

Gramstad et al. [54] recently presented the results of measurements of 27 different cytokines in 20 C1‐INH‐HAE patients and 20 controls using a 27‐plex kit (Bio‐Rad). A comparison revealed a match in the following parameters: increased levels of IL‐1ß, IL‐6, IL‐8, and MPO. However, a discrepancy was observed in other parameters: IL‐10, IL‐17A, Eotaxin, and VEGFs. The differences could be attributed to the differential proportion of C1‐INH‐HAE subtypes. They analyzed 20 C1‐INH‐HAE patients, 7 of whom had C1‐INH‐HAE type II. We analyzed 30 C1‐INH‐HAE patients, 1 of them with C1‐INH‐HAE type II. Another explanation may be that they used EDTA plasma and different assay systems (bead‐based multiplex for cytokines and singleplex ELISA for MPO). The MSD system has been described as a reliable means of quantifying levels of some cytokines that were previously undetectable by alternative assays. It has also been employed for the detection of cytokines and chemokines in difficult clinical samples [55].

IL‐17 plays a pivotal and dual role in a multitude of inflammatory and autoimmune diseases and is linked to neutrophil recruitment [57]. The divergent behavior of IL‐17A and IL‐23 was unexpected; therefore, the multiplex measurements were confirmed by single‐plex ELISA. A significant negative correlation was observed between IL‐17A and all VEGFs, as well as Tie‐2. This phenomenon has not been described before. The present study revealed elevated levels of Tie‐2 and VEGF, alongside diminished levels of IL‐17A in all AE types. In CSU, there is a paucity of research regarding serum IL‐17, with conflicting results being reported [58, 59, 60, 61], and without specifying the proportion of patients with AE with or without wheals. A preliminary report found increased IL‐17A expression in both lesional and nonlesional skin of CSU and significant improvement of the weekly urticaria activity score, but also in the severity and frequency of AE in all eight patients treated off‐label with secukinumab [62]. However, the serum levels of IL‐17A were not measured. One case report described the development of a severe urticaria with angioedema under secukinumab (anti‐IL‐17 mAb) [63]. Currently, there is little evidence for an interplay of an IL‐17‐kallikrein‐kinin system able to activate bradykinin receptors [64]. It is hypothesized that further investigation of the role of IL‐17 in angioedema is warranted.

There is mounting evidence to suggest that neutrophils may play a significant role, at least in C1‐INH‐HAE [65, 66]. The data presented herein indicate a role for NET components, MPO and MMP‐9, and eosinophil ET (EET) components like EPX. To the best of our knowledge, there are no data available regarding MMP‐9 levels in C1‐INH‐HAE or other forms of angioedema. However, as described by Grymova et al. [38], increased relative mRNA expression levels in neutrophils of C1‐INH‐HAE patients were observed in comparison to healthy controls [38]. The present findings strengthen the proposal advanced by Veszeli et al. [65] that future studies should investigate the role of NETs in C1‐INH‐HAE. In their study, MPO and pentraxin‐3 were significantly associated. In the present study, the levels of pentraxin‐3 were found to be unrelated to MPO but were instead associated with EPX. However, MPO and EPX levels were significantly associated. EPX represents a component of EETs [67, 68]. Thus, both NETs and EETs might be of interest in C1‐INH‐HAE.

In summary, the results demonstrated significant disparities, as well as some unexpected parallels, among C1‐INH‐HAE, ACEi‐AE, and Hist‐AE in comparison to healthy controls. Tie‐2, VEGFs, and C1s/C1INHc appear to play a role in all AE subtypes. The present study has identified evidence for a role of IL‐17 in AEs, as well as a role for eosinophil and neutrophil chemotactic factors and the activation of these granulocytes. The ratio of C1s/C1‐INHc with MASP‐1/C1INHc has been identified as a potential diagnostic tool to differentiate C1‐INH‐HAE from other AE subtypes. Its suitability as a biomarker of disease severity should be assessed in future studies.

### 4.1 Data Limitations and Perspectives

We acknowledge that the present study is subject to certain limitations. These include the variable time points at which blood was collected, related to the occurrence of attacks, as well as the application of on‐demand and/or prophylactic treatments. Due to the rarity of C1‐INH‐HAE (orphan disease) and the unpredictable nature of attacks, only an approximation can be made in this regard. The merits of this study are evident in the concurrent measurement of multiple analytes of the complement system and the inflammatory immune system in relatively large cohorts with different AE types in parallel. A multitude of parameters were evaluated, and the most promising that merit targeted attention in the context of the pathophysiology of angioedema were identified. In order to understand the underlying mechanisms that trigger angioedema, it would be reasonable to investigate the potential importance of complement/lectin system interactions with granulocytes in the future.

## Author Contributions

Katharina Marlies Duda collected and analyzed the data, designed the figures, and drafted the manuscript. Manuela Gehring assisted with the ELISA assay and the corresponding data analysis. Bettina Wedi conceptualized the study, assisted with data analysis, created Figure 8, and edited figures and the manuscript. All authors contributed to the completion and revision of the manuscript.

## Disclosure

The content of this publication does not necessarily reflect the views or policies of the Department of Health and Human Services, nor does it mention the trade names, commercial products, or organizations that imply endorsement by the US Government.

## Ethical Statement and Consent

The manuscript under consideration does contain human studies. The local Ethical Committee approved the studies, and informed consent of all participating subjects was obtained. The ethic votum number and informed consent procedure were incorporated into the method section of the manuscript text. All studies Ethics committee approval number 7409 of 26.04.2017, extended 28.07.2021 by Hannover Medical School, Hannover, Germany. Informed written consent was obtained from each patient.

## Conflicts of Interest

Katharina Marlies Duda reports support for attending meetings and/or travel from Biocryst, CSL Behring, Takeda, and participation on a one‐day advisory board of Takeda; all outside the manuscript. Manuela Gehring declares no conflicts of interest. Dr. Wedi reports personal honoraria for lectures, presentations, or educational events from ALK‐Abéllo, Bencard, CSL Behring, Novartis, Takeda, and support for attending meetings and/or travel from CSL Behring, Novartis, Takeda, and participation on one‐day advisory boards from Biocryst, CSL Behring, Kalvista, Novartis, Sanofi‐Aventis, Takeda; all outside the manuscript.

## Data Availability

The data that support the findings of this study are available from the corresponding author upon reasonable request.
